# Analytical results on the Beauchemin model of lymphocyte migration

**DOI:** 10.1186/1471-2105-14-S6-S10

**Published:** 2013-04-17

**Authors:** Johannes Textor, Mathieu Sinn, Rob J de Boer

**Affiliations:** 1 Theoretical Biology & Bioinformatics, Utrecht University, 3508 TC Utrecht, The Netherlands; 2 Institute for Theoretical Computer Science, University of Lübeck, 23562 Lübeck, Germany; 3 IBM Research, Mulhuddart, Dublin 15, Ireland

## Abstract

The Beauchemin model is a simple particle-based description of stochastic lymphocyte migration in tissue, which has been successfully applied to studying immunological questions. In addition to being easy to implement, the model is also to a large extent mathematically tractable. This article provides a comprehensive overview of both existing and new analytical results on the Beauchemin model within a common mathematical framework. Specifically, we derive the motility coefficient, the mean square displacement, and the confinement ratio, and discuss four different methods for simulating biased migration of pre-defined speed. The results provide new insight into published studies and a reference point for future research based on this simple and popular lymphocyte migration model.

## Background

A unique property of the immune system is that it mainly consists of constantly moving cells. Technological progress has facilitated the study of lymphocyte migration at increasingly higher resolutions, culminating in the application of two-photon microscopy to tracking single lymphocytes in the living animal [1-3]. Accompanying this progress, mathematical and computational models of lymphocyte migration have been developed and applied to both qualitative and quantitative questions. These models can be roughly categorized as follows according to their level of detail: Whole-population models, usually formulated using ordinary or partial differential equations, do not distinguish between individual cells, but treat cell subpopulations as continuous quantities. Particle-based models do consider each cell individually, but treat cells as freely moving particles without mass, volume, and shape. Finally, whole-cell models such as the Cellular Potts Model [4] explicitly represent the cell and its interaction with the environment. In the present paper, we analyze a particle-based model that was introduced by Beauchemin, Dixit, and Perelson [5]. Throughout the paper, we refer to this model as the "Beauchemin Model".

Particle-based approaches such as the Beauchemin model are used for studying questions where a whole-population approach does not provide sufficient information, but a whole-cell model is not necessary, not feasible for computational reasons or would require too many assumptions on unknown parameters. For instance, Grigorova et al. [6] and ourselves [7] recently used the Beauchemin model to study the transit of T cells through a lymph node in the absence of antigen, and we used it to determine the amount of directional bias that could be detected in random T cell migration using contemporary two-photon imaging techniques [7].

The Beauchemin model is defined by three parameters - a speed *v*_free_, a time interval *t*_free_, and another time interval *t*_pause_. The model describes a particle moving freely in a three-dimensional space according to the following rules: The particle starts at a fixed position and turns in a random direction (more precisely, a direction is sampled from a uniform distribution on the unit sphere). The turn takes a fixed time *t*_pause_, during which the cell does not move. This reflects the time it takes to "displace the internal structure which brings about motion" [4]. Then, the cell moves in the chosen direction with velocity *v*_free _for a fixed time *t*_free_, after which it stops and restarts the process.

The main distinguishing characteristic of the model is the introduction of the pause phase. In this sense, it can be seen as a generalization of the "ideal chain" model of polymer physics [8], in which a chain of rigid rods of fixed length *ℓ *is considered that are freely jointed to each other. This is mathematically equivalent to the Beauchemin model if we set *v*_free _· *t*free = *ℓ *and *t*_pause _= 0. In particular, analytical results e.g. on the mean square displacement of the ideal chain [8] are directly transferable to this case. As we shall see later on in this paper, for some quantitative properties even the more general case with *t*_pause _≥ 0 can be reduced to the ideal chain model.

This paper is structured as follows: In the upcoming section, we begin by formulating a mathematical framework for our analysis, which explicates sufficiently but not overly general notions of random walks and Brownian motion. We deliberately include a fairly large amount of detail, including proofs, in order to present our results in a self-contained fashion. Thereafter, we present analytical results about random motion in the Beauchmin model, and discuss four different ways for simulating biased migration with pre-defined speed. We wrap up with concluding remarks.

We point out that around half of the results discussed in this paper originate from our recent study that used the Beauchemin model [7]. We chose to integrate these results into the present paper in rewritten and extended form because they were formerly only published in condensed form as supporting online material. In this manner, we hope to provide a coherent account of all available analytical results, which we anticipate to serve as a useful reference for future users of the model. We mark results that have appeared earlier in our previous study or elsewhere (e.g. Proposion 10) by giving the appropriate reference in proposition or theorem statements, while propositions or theorems stated without such a reference (e.g. Proposion 12) have, to our knowledge, not been published before.

## Mathematical foundations

In this section, we outline the basic mathematical framework that we use to describe stochastic cell motion and, later on, the Beauchemin model. A similar, but slightly less general framework (based on random walk on a lattice) is used in Berg's textbook *Random Walks in Biology *[9], while a much more general account of random walks than given here is usually presented in probability textbooks (e.g., [10]). At the end of this section, we will outline the connection of this random walk to the corresponding macroscopic partial differential equation model, which is the convection-diffusion-equation.

First of all, we introduce some notational conventions. We denote the expectation of a random variable *ξ *by E[*ξ*], its variance by Var(*ξ*) = E [(*ξ *− E[*ξ*])^2^] and its probability density function by *f*_ξ_. Elementary random variables are written in lowercase Greek letters (usually *ξ_i_*) while derived random variables that are functions of elementary random variables are written in capital Latin letters. All random variables are assumed to be continuous and real-valued. Mean and variance of random variables will also be written as *μ *and *σ*^2^, if the referenced variable is clear from context.

### Observed tracks and associated measures

The Beauchemin model is usually validated against, or used to generate, cell tracking data as it would be recorded in a two-photon microscopy experiment. As we will see later, there can be subtle differences between the real underlying state and the observed state of a cell. We thus make this difference explicit in our analysis by means of the following definitions for cell tracking terminology.

**Definition 1 **(Cell track). *A d-dimensional *cell track *is a finite sequence*

(x(1),t(1)),…,(x(k),t(k))

*consisting of positions *$x\left(1\right)\;,\ldots ,\;x\left(k\right)\in {\mathbb{R}}^{d}$*and increasing time points *t(1),…,t(k)∈ℝ.

The time indices are written in brackets to indicate that the positions are discrete-time observations of an underlying continuous motion process. For population-based cell track analysis, it is common to align a set of tracks to a common starting point.

**Definition 2 **(Aligned cell track sets). *For a cell track T *= (*x*(1), *t*(1)) , ..., (*x*(*k*), *t*(*k*)), *the track that results from subtracting the first element from all elements in the sequence, i.e*.

$$T_{0}=\left(0,0\right),\left(x\left(2\right)-x\left(1\right),t\left(2\right)-t\left(1\right)\right)\;,....,\;\left(x\left(k\right)-x\left(1\right),t\left(k\right)-t\left(1\right)\right)$$

*is called the zero-aligned version of T *.

Throughout the paper, we will assume that all cell track sets are zero-aligned.

**Definition 3 **(Mean displacement and mean square displacement). *Let x*_1_(*t*) , ..., *x_n _*(*t*) *be the positions of n zero-aligned cell tracks at some fixed time t. Then the mean displacement D*(*t*) *for S is defined by*

$$D\left(t\right)=\frac{\left||x_{1}\left(t\right)||+\;\cdots \;+||x_{n}\left(t\right)|\right|}{n}$$

*where *|| · || *is the d-dimensional euclidian norm or vector length, and the mean square displacement D*^2^(*t*) *is defined by*

$$D^{2}\left(t\right)=\frac{||x_{1}\left(t\right)|{|}^{2}+\;\cdots \;+||x_{n}\left(t\right)|{|}^{2}}{n}$$

Beauchemin et al. [5] estimated the three parameters *v*_free_, *t*_free _and *t*_pause _of their model by fitting it to mean displacement data: For each combination of parameters, 10^6 ^simulations of the model were run, and the mean displacement data was recorded and averaged over all simulations. The resulting mean displacement curve was compared to real data from several publications. The parameter triplets were ranked according to their squared sum of errors to the real data (see Table 1). It was found that the values *t*_free _= 2.0 min, *t*_pause _= 0.5 min and *v*_free _= 18.8 *μ*m/min gave the best agreement to the real data. These values are biologically reasonable and similar to those estimated by Miller et al. [1].

**Table 1:** Different parameters can lead to similar motility coefficients.

**Rank**	***t*_pause_**	***t*_free_**	***v*_free_**	***M***	**SSR**
	**min**	**min**	***μ*m/min**	***μ*m^2^/min**	
1st	0.5	2.0	18.8	94.25	1.00
2nd	0.5	2.5	16.6	95.68	1.01
3rd	0.25	2.0	17.6	91.78	1.02
4th	1.5	1.5	26.1	92.89	1.03
5th	0.75	1.5	23.8	94.41	1.03

Parameter triplets of the 3D cell random walk model that were found to best fit *in vivo *data by Beauchemin et al. [5] along with their corresponding motility coefficients as per equation (2). Beauchemin et al. calculated sums of squared residuals (SSR) to a set of *in vivo *mean displacement data from four publications [24,1,26]. The SSR values shown here are normalized to the best ranking triplet.

### Microscopic random walk with bias

In this section, we will define the notion of a one-dimensional random walk used in this paper and derive its relation to a one-dimensional convection-diffusion process. These concepts can be easily generalized to higher dimensions under the assumption that the dimensions are pairwise uncorrelated, while stochastic independence in the strict sense is not necessary. This assumption holds for the particular random walks we analyze in this paper.

**Definition 4 **(One-dimensional random walk [9,11]). *Let ξ_i_*, i∈ℕ, *be independent, identically distributed real-valued random variables with mean μ and variance σ*^2^. *Then the sequence *(*S_t_*), i∈ℕ, *defined by*

$$S_{t}=\overset{t}{\underset{i=1}{\sum }}\xi_{i}$$

*is called a random walk with step bias μ and step variance σ*^2^. *If μ *=0, (*S_t_*) *is called an unbiased random walk*.

Intuitively, the sequence (*S_t_*) is the random walk trajectory and the *ξ_i _*are the individual steps. The above definition is a slightly generalized version of the commonly used entirely discrete random walk with fixed steps to the left and the right: We assume that each step is sampled from a (possibly continuous) random variable with finite first and second moments. The following corollary provides an important characteristic of this random walk:

**Corollary 5 **(Mean and variance of a random walk). *For a random walk *(*S_t_*) *as given by Definition 4*,

$$E\left[S_{t}\right]=t\mu \;and\;Var\left(S_{t}\right)=t\sigma^{2}.$$

*Proof*. Since the *ξ_i _*are independent and thus uncorrelated, this is a direct consequence of the linearity of variance and expectation.    □

**Figure 1:**
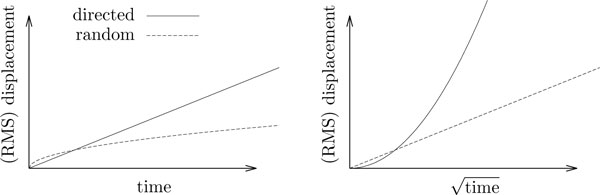
**Directed vs. random motion**. Traveled distance (directed movement) and root mean square displacement (random movement) of directed and random motion plotted as functions of time (left) and square root of time (right). On the left hand side, directed motion yields a straight line, with a slope given by its *speed*. On the right hand side, random motion yields a straight line, with a slope given by its *diffusion coefficient *(also called *motility coefficient *in this paper).

If the random walk is unbiased, Var(*S_t_*) can be geometrically interpreted as the expected mean square squared displacement of the particles at time *t *(Definition 3). This leads to an important characteristic of the unbiased random walk: In a particle ensemble, the particles will go nowhere on average, and their expected mean square displacement is linear in time. This characterization is often used in the immunological literature [2] to convey the difference between a random walk versus directed migration, where the *root *mean square displacement is linear in time (Figure 1).

For sufficiently long observations, we do not only know the variance and mean of a random walk, but can furthermore obtain a good approximation of the entire particle distribution from the central limit theorem.

**Theorem 6 **(Central limit theorem [9,10]). *Let *(*S_t_*) *be a random walk as given in Definition 4. Then as t *→ ∞*, the distribution of the random variable*

$$Z_{t}=\frac{S_{t}-t\mu }{\sqrt{t}}$$

*converges to a normal distribution with mean *0 *and variance σ*^2 ^.

Thus, after a sufficiently long time, the distribution of the random walk (*S_t_*) at fixed times is approximately normal. Of course, the terms "sufficiently long" and "approximately normal" are rather vague. In the context of intravital two-photon imaging, which is limited to a finite, often axially thin imaging region, we can usually not safely assume that we are in the range of validity of the normal distribution approximation: We are observing only a few "steps" (prolonged periods of rather persistent motion followed by short pauses) of a random walk. Beltman et al. [3] and ourselves [7] have discussed the implications of this technical limitation for detecting directional bias and estimating the motility coefficient.

We now generalize the random walk to higher dimensions.

**Definition 7 **(Multi-dimensional random walk [7]). *Let *d∈ℕ. *Suppose *$\left(\xi_{i}^{\left(1\right)},\ldots ,\xi_{i}^{\left(d\right)}\right)$, i∈ℕ*are independent, identically distributed *${\mathbb{R}}^{d}$-*valued random vectors with *$\xi_{i}^{\left(1\right)},\ldots ,\xi_{i}^{\left(d\right)}$*pairwise uncorrelated*, $E\left[\xi_{i}^{\left(1\right)}\right]=\mu^{\left(1\right)},\ldots ,E\left[\xi_{i}^{\left(d\right)}\right]=\mu^{\left(d\right)}$*and *$Var\left(\xi_{i}^{\left(1\right)}\right)=\ldots =Var\left(\xi_{i}^{\left(d\right)}\right)=\sigma^{2}$. *Then the sequence *(*S_t_*), t∈ℕ, *defined by*

$$S_{t}=(S_{t}^{\left(1\right)},\ldots ,S_{t}^{\left(d\right)}=\overset{t}{\underset{i=1}{\sum }}\left(\xi_{i}^{\left(1\right)},\ldots ,\xi_{i}^{\left(d\right)}\right)$$

*is called a d-dimensional random walk with step bias *(*μ*^(1)^, ..., *μ*^(d)^) *and step variance σ*^2^. *If μ*^(1) ^= ... = *μ*^(d) ^= 0*, then *(*S_t_*) *is called an unbiased d-dimensional random walk*.

The following theorem gives the limit distribution of suitably scaled multi-dimensional random walks within our framework.

**Theorem 8 **(Multi-dimensional central limit theorem [7]). *Let *(*S_t_*) *be a d-dimensional random walk as given in Definition 7. Then as t *→ ∞*, the distribution of the random vector*

$$Z_{t}=\frac{\left(S_{t}^{\left(1\right)}-t\mu^{\left(1\right)},\ldots ,\;S_{t}^{\left(d\right)}-t\mu^{\left(d\right)}\right)}{\sqrt{t}}$$

converges to a d-dimensional normal distribution with zero means and covariance matrix

$$\sum \;=\;\left(\begin{array}{ccc} \sigma^{2} & 0 & 0\\ 0 & \sigma^{2} & 0\\ 0 & 0 & \sigma^{2} \end{array}\right)\;.$$

*Proof*. Let $a_{1},\ldots ,\;a_{d}\in \mathbb{R}$. According to the Cramér-Wold Theorem, it suffices to show that $\left(a_{1}\left(S_{t}^{\left(1\right)}-t\mu^{\left(1\right)}\right)+\;\ldots \;+a_{d}\left(S_{t}^{\left(d\right)}-t\mu^{\left(d\right)}\right)\right)/\sqrt{t}$ converges in distribution to a normal random variable with zero mean and variance $\left(a_{1}^{2}+\ldots +a_{d}^{2}\right)\sigma^{2}$ (see [12]). Note that the random variables $a_{1}\xi_{i}^{\left(1\right)}+\;\ldots \;+a_{d}\xi_{i}^{\left(d\right)}$, i∈ℕ, are independent, identically distributed and have mean *a*_1_*μ*^(1) ^+ *..*. + *a_d_μ*^(*d*)^. Furthermore, since $\xi_{i}^{\left(1\right)},\ldots ,\;\xi_{i}^{\left(d\right)}$ are pairwise uncorrelated,

$$\text{Var}\left(a_{1}\xi_{i}^{\left(1\right)},\ldots ,\;a_{d}\xi_{i}^{\left(d\right)}\right)=\left(a_{1}^{2}+\;\ldots \;+a_{d}^{2}\right)\sigma^{2}.$$

Hence, the result follows from the central limit theorem for one-dimensional random walks.    □

The central limit argument shows that even complex cell migration processes might have a simple description when observed for a sufficiently long time: The diffusion coefficient is a function of all "microscopic" cell motility parameters such as persistence time, speed, turning angle distributions and so on. In the scaling limit, this single coefficient describes an unbiased random walk completely. Therefore, it is more common to describe random walk processes on such timescales in terms of a differential equation instead of a stochastic process. This can be done using a *scale transition*, as we will explain below.

### The convection-diffusion equation

The random walk (*S_t_*) introduced in Definition 4 is a plausible microscopic model for the position of particles. However, if the positions are observed on large time scales (e.g., only every 20th state of the random walk can be observed due to a limited temporal resolution), then a macroscopic continuous model can be used to describe the migration. This continuous model no longer depends on the detailed characteristics of the microscopic model such as the turning angle distribution or persistence length (i.e., the precise shape of the *ξ_i_*).

Mathematically, we construct the continuous model as follows: For n∈ℕ let (*Z*_n_(*t*)) with *t *≥ 0 be given by

(1) $$Z_{n}\left(t\right)=\frac{1}{\sqrt{n}}\overset{\left\lfloor tn\right\rfloor }{\underset{i=1}{\sum }}\left(\xi_{i}-\mu \right)+t\mu .$$

We write the time index *t *in brackets in order to emphasize that the processes are given in continuous time. The process (*Z_n _*(*t*)) is obtained by observing *n *steps of the random walk (*S_t_*) in every time interval (*t *− 1, *t*] with t∈ℕ. It is scaled such that E [*Z_n _*(*t*)] = E [*S_t_*] = *tμ *and Var(*Z_n_*(*t*)) = Var(*S_t_*) = *tσ*^2 ^for every t∈ℕ. Between two successive steps, the process is constant.

Let (*Z*(*t*)), *t *≥ 0, denote the limiting process obtained as *n*, the number of steps per time interval, tends to infinity. According to the Functional central limit theorem (sometimes also referred to as *Donsker's Theorem*, see [12]), (*Z*(*t*)) has the following two properties:

• Any finite-dimensional distribution of (*Z*(*t*)) is Gaussian.

• E[*Z*(*t*)] = *tμ *and Cov(*Z*(*s*), *Z*(*t*)) = min(*s*, *t*)*σ*^2 ^for *s*, *t *≥ 0.

A process with these properties is called *Brownian motion *with *drift μ*. If *t>*0, then *Z*(*t*) has a probability density *ϕ*(*x*, *t*) given by

$$\phi \left(x,t\right)=\frac{1}{\sqrt{2\pi t\sigma }}\;\text{exp}\;\left(-\frac{{\left(x-t\mu \right)}^{2}}{2t\sigma^{2}}\right)$$

for x∈ℝ.

As one can easily verify, this probability density function solves the following well-known differential equation for the initial condition *ϕ*(*x*, 0) = *δ*(*x*), where *δ *denotes the Dirac delta distribution.

**Theorem 9 **(Convection-diffusion equation [11,13]). *Let C *= *μ and M *= *σ*^2^/2. *Then*

$$\frac{\partial }{\partial t}\phi \left(x,t\right)=\left[-C\frac{\partial }{\partial x}\;+M\frac{\partial^{2}}{\partial x^{2}}\right]\;\;\phi \left(x,t\right).$$

Taken the initial condition given above, the solution of the convection-diffusion-equation exists and is unique [13].

As the sum of independent Brownian motions is again a Brownian motion, the convection-diffusion equation describes the dynamics of single diffusing particles as well as of ensembles of particles carrying out motions independently: Assuming the ensemble is large enough such that stochastic perturbations can be ignored, the convection-diffusion-equation describes the evolution of the particle concentration over time. This scaling argument justifies the use of the convection-diffusion equation for simulating large cell populations on timescales that are substantially longer than the rhythmicity of the cell migration process. For example, we used the convection-diffusion equation to simulate the transit of T cells through the lymph node, which takes approximately half a day [7].

Note that in the context of molecular motion, the quantity *M *is usually called *diffusion coefficient*. Throughout this paper we will use the term *motility coefficient *to indicate that we are talking about particles that are substantially larger than molecules; this is common in the immunological literature since Miller et al.'s early work [1].

Similarly as for the discrete model, the continuous model can be generalized to multiple dimensions. In this case, (*Z_n _*(*t*)) is obtained by observing *n *steps of a *d*-dimensional random walk (*S_t_*) in every time interval of unit length. For *n *tending to infinity, the Functional central limit theorem yields convergence to a *d*-dimensional Brownian motion with independent components and convection coefficients *C*^(1) ^= *μ*^(1) ^, ..., *C*^(*d*) ^= *μ*^(*d*)^. The motility coefficient is equal to *M *= *dσ*^2 ^/2, where *d *is the number of observed dimensions.

### Summary

As a microscopic model for stochastic motility of particles, we have first introduced one-dimensional random walks. According to this model, particles move by taking independent and identically distributed steps at equidistant discrete time points. Therefore, just like in the Beauchemin model, our framework does not consider persistence to last longer than the duration of a single step; the model is fully characterized by the absolute time between successive steps and the distribution of steps. Important characteristics of the step distribution are the step bias *μ *and the step variance *σ*^2^. The random walk is called unbiased if *μ *= 0, and biased, otherwise. If we observe many particles carrying out simultaneous unbiased random walks, the particles will go nowhere on the average, but they will spread out increasingly, their distribution being approximately normal. The model can be easily generalized to *d *dimensions if we assume that the steps are dimension-wise uncorrelated (not necessarily statistically independent). Essentially, we can then treat the *d *dimensions like *d *separate one-dimensional random walks.

**Figure 2:**
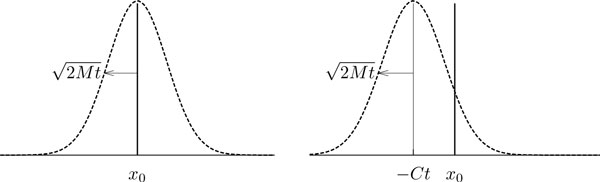
**Position distributions for biased random walks**. Expected distribution of zero-aligned cell displacements measured in one dimension in the case of an unbiased random walk with motility coefficient *M *(left) and a biased random walk with convection coefficient *C *(right) as per theorem 9. In both cases, the distribution is Gaussian with standard deviation $\sqrt{2tM}$. In the biased case, the mean of the distribution moves away from the origin with time.

On the macroscopic scale, (biased) random walks can be approximated by Brownian motion (with drift). In this case the motion of particles is described sufficiently well by only two parameters, namely the *motility coefficient M *= σ^2 ^/2 and the *convection coefficient C *= *μ*. The motility coefficient *M *quantifies the random component of a biased random walk, while the convection coefficient *C *(which can be interpreted as a velocity) characterizes the directed component, if there is any (Figure 2). Convergence to Brownian motion can also be obtained for much more general random walk models using appropriate central limit theorems. However, the particular random walk chosen here is general enough for our purpose of analyzing the Beauchemin model.

## The Beauchemin model

In this section, we analytically derive three quantitative characteristics of the particular random walk defined by the Beauchemin model, namely the motility coefficient, the mean square displacement, and the confinement ratio.

### Motility coefficient

The fact that the model is three-dimensional might seem to complicate our analysis. However, fortunately, it is possible to decouple the dimensions of the model, as will be shown in the following. The upcoming result was stated by ourselves [7] and, more recently, by Donovan and Lythe [14].

**Proposition 10 **(Motility Coefficient of the Beauchemin model [7,14]). *Let *$\left(\xi_{i},v_{i},\zeta_{i}\right)\in {\mathbb{R}}^{3}$, i∈ℕ, *be independent random vectors distributed uniformly on a sphere with radius r. Set *$\left(X_{t},\;Y_{t},\;Z_{t}\right)=\sum_{i=1}^{t}\left(\xi_{i},v_{i},\;\zeta_{i}\right)$. *Then X_t_, Y_t _and Z_t _are unbiased random walks as defined in 4, and as t *→ ∞*, the distribution of $\left(X_{t},Y_{t},Z_{t}\right)/\sqrt{t}$ converges to a 3-variate normal distribution with zero mean and covariance matrix*

$$\sum \;=\;\left(\begin{array}{ccc} \sigma^{2} & 0 & 0\\ 0 & \sigma^{2} & 0\\ 0 & 0 & \sigma^{2} \end{array}\right),\;\sigma^{2}=r^{2}/3.$$

*Proof*. The key ingredients to the proof are

(*i*) *ξ_i_*, *υ_i_*, *ζ_i _*are uniformly distributed on [−*r*, *r*].

(*ii*) Cov(*ξ_i_*, *υ_i_*) = Cov(*ξ_i_*, *ζ_i_*) = Cov(*υ_i_*, *ζ_i_*) = 0.

In order to establish (*i*), note that *P *(*ξ_i _< h*), i.e., the probability that *ξ_i _*lies below the height *h *in the sphere, is proportional to the part of the sphere's surface area lying below the plane *ξ_i _*= *h*. The surface of a spherical cap is proportional to its height, and thus *ξ_i _*must be uniformly distributed on [−*r*, *r*] (see [15]). By isotropy, the same arguments hold for *υ_i _*and *ζ_i_*. The statement (ii) on pairwise uncorrelatedness is obtained by (*ii*) is obtained by observing that (*ξ_i_*, *υ_i_*) is uniformly distributed on a circle with radius *r *− |*z*| given that *ζ_i _*= *z*, and hence Cov(*ξ_i_*, *υ_i_*) = 0.

Now, (*i*) implies E [*ξ_i_*] = E [*υ_i_*] = E [*ζ_i_*] = 0, and hence (*X_t_*), (*Y_t_*) and (*Z_t_*) are unbiased random walks. The statement on the asymptotic distribution of (*X_t_*, *Y_t_*, *Z_t_*) follows by (*ii*) together with Theorem 8, and the fact that a random variable with uniform distribution on [−*r*, *r*] has variance *r*^2^/3.    □

Hence, particles in the Beauchemin model behave exactly like the superposition of three uncorrelated random walks with uniformly distributed steps.

### Mean square displacement

Putting together our result from the last section with the relationship between the motility coefficient *M *and the dimension-wise step variance *σ*^2 ^of a random walk, we obtain the following relation between the diffusion coefficient and the microscopic parameters:

(2) $$M=\;\frac{{\left(v_{\text{free}}\times t_{\text{free}}\right)}^{2}}{6\left(t_{\text{free}}+t_{\text{pause}}\right)}$$

The corresponding diffusion coefficients for the 5 best-fitting parameter triplets determined by Beauchemin et al. are given in Table 1. It turns out that all the best-fitting triplets have very similar motility coefficients. On the other hand, the microscopic motility parameters vary quite a lot: For example, the pause time of the 4th best fitting triplet is 5 times longer than that of the 3rd best fitting triplet. The triplets were ranked by Beauchemin et al. according to the sum of squared residuals (SSR) to the experimental data. However, the SSR of the 5th best fitting triplet is only less than 4% higher than that of the best fitting triplet. Our upcoming analysis will provide some insight why this occurs.

The next two propositions will yield a precise characterization of the *variance *- and with it the mean square displacement - of the position of single particles and ensembles of observed particles in the Beauchemin model. It is mathematically more convenient to analyze the mean square displacement rather than the mean displacement because, as we have shown above, the dimensions can be decoupled. For mean displacement, this does not hold, and we would have to take into account the stochastical dependence between dimensions.

We start out with the variance of the position of one particle that starts its random walk at time *t *= 0. For simplicity, we will derive the following equations assuming that one dimension is observed. Due to the independence of the dimensions discussed above, multi-dimensional versions of these results are obtained simply by multiplying the derived quantity (i.e., mean square displacement or confinement ratio) with the number of observed dimensions.

**Proposition 11 **(Expected square displacement of single particles in the Beauchemin model). *A particle starts at time t *= 0 *in position x*(0) = 0 *to carry out a random walk as defined by the Beauchemin model with fixed parameters t_pause_,t_free_,v_free_. Let x*(*t *+ *τ*) *denote the position of the particle observed in one dimension at time t *+ *τ where t is an integer multiple of t_free _*+ *t_pause _and *0 ≤ *τ < t_free _*+ *t_pause_. Then the expected square displacement of the particle is given by:*

$$E\;\left[D^{2}\left(t+\tau \right)\right]=Var\left(x\left(t+\tau \right)\right)=2Mt+\frac{v_{free}^{2}}{3}{\left(\text{max}\left(\tau -t_{pause},0\right)\right)}^{2}$$

*with M as defined in equation (2)*.

*Proof*. The term 2*Mt *follows directly from Proposition 10 and the linearity of variance (see also Corollary 5). Thus we continue with *t *= 0. Then we have from Proposition 10 that the position at time *t*_free _+ *t*_pause _is uniformly distributed on the interval [−*v*_free_*t*_free_, *v*_free_*t*_free_], having variance 2*M*(*t*_free _+ *t*_pause_).

For *τ *≤ *t*_pause_, the particle's position does not change, hence its variance is equal to 0. For *τ > t*_pause_, the position is uniformly distributed on an interval with a size proportional to *τ *− *t*_pause_. Hence the variance has quadratic form *y *= *a*(*τ *− *t*_pause_)^2^. Inserting *τ *= *t*_free _+ *t*_pause_, we obtain

$$2M\left(t_{\text{free}}+t_{\text{pause}}\right)=a\;t_{\text{free}}^{2}$$

Inserting the definition of *M *from above, this results in

(3) $$a=\frac{{\left(v_{\text{free}}\right)}^{2}}{3}$$

Combining the cases *τ *≤ *t*_pause _and *τ > t*_pause_, we arrive at the proposition.    □

**Figure 3:**
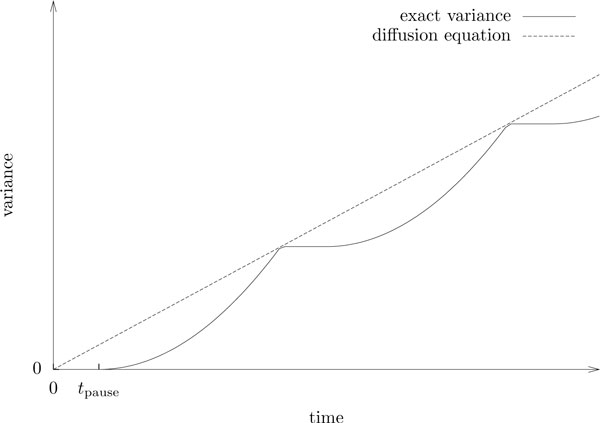
**The Beauchemin model and the diffusion equation**. The expected square displacement (or position variance) of a single particle along with the approximation by the diffusion equation.

A plot of the expected square displacement shows a series of iterated pulses that approaches the linear progression described by the diffusion equation from below (see Figure 3). However, we will most likely not see such phasic behaviour if we take the average of several observed particles, since the particles will in general not be synchronized and the pulses that are caused by the free runs between turns will average out. Consequently, the next step in our analysis is to derive the expected mean square displacement for a particle ensemble. We do this by assigning to each observed particle a *phase*, which reflects the particle's state at the time when we start our observation. Then we average over all possible phases.

**Proposition 12 **(Empirical mean square displacement of the Beauchemin model). *Set t_run _*= *t_free _*+ *t_pause_. A particle starts at a uniformly distributed random time *−*t*_0_, −*t_run _<*−*t*_0 _≤ 0 *in some position to carry out a random walk as defined by the Beauchemin model with fixed parameters t_pause_, t_free_, v_free_. Let x(t)∈ℝ denote the position of this particle observed in one dimension at time t, where we choose the coordinate system such that x*(0) = 0. *Then the expected square displacement of the particle at time t is given by*

$$\begin{array}{llll} E\left[D^{2}\left(t\right)\right] & =\frac{1}{t_{run}}\int_{-t_{run}}^{0}\;Var\left(x\left(t-t_{0}\right)-x\left(t_{0}\right)\right)\;dt_{0}\; & & \;\\ & =2Mt-2Mt_{free}\times \;\left\{\begin{array}{cc} \frac{1}{3} & t\geq t_{free}\\ \frac{1}{3}{\left(\frac{t}{t_{free}}\right)}^{3}-{\left(\frac{t}{t_{free}}\right)}^{2}+\left(\frac{t}{t_{free}}\right) & t<t_{free} \end{array}\right).\; & & \;\\ & \; & \end{array}$$

**Figure 4:**
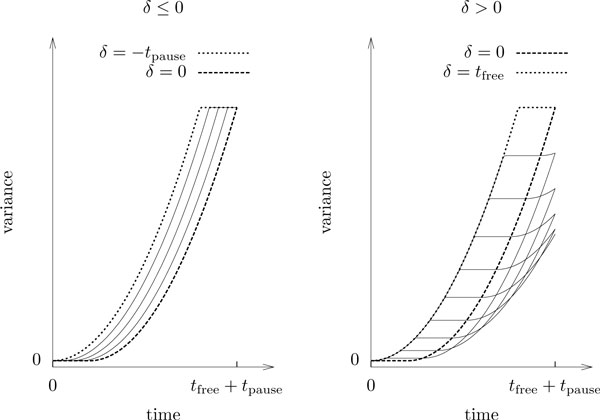
**Effect of the phase parameter on the variance**. The plots illustrate how the phase parameter *δ *affects the variance of a single observed cell in the Beauchemin model. The expected variance for a (large) ensemble of observed particles is obtained by averaging over all values of *δ*.

*Proof*. See Figure 4 for an illustration of how the parameter *δ *affects the variance of the observed particle position during the initial observation time. For *δ *≤ 0, the cell is resting at the beginning of observation time and remains paused for time *t*_pause _− *δ*. For *δ >*0, the cell is moving at the beginning of observation time and keeps moving for time *δ*. Let us write Var(*S_δ_*(*t*)) for the position variance of a particle with phase shift *δ*.

Denote by *τ*_0 _the time that the particle was already moving when we started observing it. Hence,

$$\hat{x}\left(t\right)=x\left(t+\tau_{0}\right)-x\left(\tau_{0}\right)$$

where $\hat{x}\left(t\right)$ denotes our observed position. We are interested in the variance $Var\left[\hat{x}\left(t\right)\right]$, for which the following holds:

(4) $$\text{Var}\left[\hat{x}\left(t\right)\right]$$

(5) $$=\text{Var}\left[x\left(t+\tau_{0}\right)-x\left(\tau_{0}\right)\right]$$

(6) $$=\text{Var}[x(t+\tau_{0})]+\text{Var}[x(\tau_{0})]-2\text{Cov}[x(t+\tau_{0},\;x\left(\tau_{0}\right)]$$

What we are deriving is the expectation of expression 6, which is a function of the random variable *τ*_0_. Due to the markov property of the random walk, only the last observed step matters and we can take *τ*_0 _as uniformly distributed over [0, *t*_free _+ *t*_pause_). Thus,

$$\text{E}\left[\text{Var}\left[\hat{x}\left(t\right)\right]\right]=\frac{1}{t_{\text{free}}+t_{\text{pause}}}\int_{\tau =0}^{t_{\text{free}}+t_{\text{pause}}}\text{Var}\left[x\left(t+\tau_{0}\right)\right]-\text{Var}\left[x\left(\tau_{0}\right)\right]d\tau_{0}$$

We start by integrating the two variance terms. Some algebra yields

$$\begin{array}{llll} & \;\frac{1}{t_{\text{free}}+t_{\text{pause}}}\int_{\tau_{0}=0}^{t_{\text{free}}+t_{\text{pause}}}\text{Var}\left[x\left(t+\tau_{0}\right)\right]+\text{Var}\left[\tau_{0}\right]d\tau_{0}\; & & \;\\ & =\frac{{\left(v_{\text{free}}t_{\text{free}}\right)}^{2}}{3\left(t_{\text{free}}+t_{\text{pause}}\right)}\left(\left(t+\frac{t_{\text{free}}}{3}\right)+\frac{t_{\text{free}}}{3}\right)\; & & \;\\ & =2M\;\left(\left(t+\frac{t_{\text{free}}}{3}\right)+\frac{t_{\text{free}}}{3}\right)\; & & \;\\ & \; & \end{array}$$

where *M *is the motility coefficient as defined in equation 2. For the covariance term it can be shown by some case distinctions that

$$\begin{array}{llll} & \;\frac{1}{t_{\text{free}}+t_{\text{pause}}}\int_{\tau_{0}=0}^{t_{\text{free}}+t_{\text{pause}}}\text{Cov}\left[x\left(t+\;\tau_{0}\right),\;\tau_{0}\right]d\tau_{0}\; & & \;\\ & =M\text{min}\left(t,t_{\text{free}}\right)+M\underset{=0\;\text{for}\;t\geq t_{\text{free}}}{\underset{︸}{\left(\frac{\text{min}{\left(t,t_{\text{free}}\right)}^{2}}{t_{\text{free}}}+\frac{\text{min}\left(t,t_{\text{free}}\right)3}{3\overset{2}{\underset{\text{free}}{t}}}+\frac{2t_{\text{free}}}{3}\right)}}\; & & \;\\ & \; & \end{array}$$

Combining the variance and covariance terms as per expression 6, we obtain the claimed identity.    □

**Figure 5:**
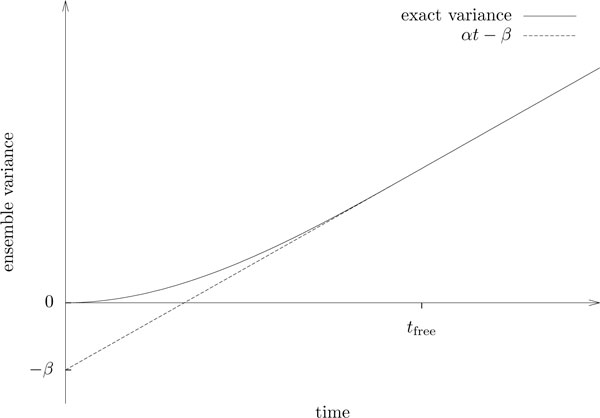
**Forming the population average**. Expected mean square displacement of observed particle ensembles as of Proposition 12. The pulses of the individual variances (see Figure 4) average out, and the exact expression converges quickly to a linear form with slope *α *= 2*M *and intercept *β *= 2*Mt*_free_/3. One degree of freedom from the individual variance is lost due to the averaging process.

As illustrated in Figure 5, the empirical mean square displacement from time *t*_free _onwards is thus perfectly described by the linear equation

(7) $$\text{E}\;\left[D^{2}\left(t\right)\right]=\alpha t+\beta$$

with slope

(8) $$\alpha =2M=\frac{{\left(v_{\text{free}}\right)}^{2}{\left(t_{\text{free}}\right)}^{2}}{3\left(t_{\text{free}}+\;t_{\text{pause}}\right)}$$

and intercept

(9) $$\beta =-\frac{2t_{\text{free}}}{3}M=-\frac{{\left(v_{\text{free}}\right)}^{2}{\left(t_{\text{free}}\right)}^{3}}{9\left(t_{\text{free}}+t_{\text{pause}}\right)}.$$

This is an important difference to other models of mean square displacement such as Fürth's equation, which is frequently applied to cell migration data [16,17]. This equation reads

(10) $$\text{E}\;\left[D^{2}\left(t\right)\right]=2M\left(t-P\left(1-e^{-t/P}\right)\right)$$

where *P *is a parameter called the *persistence length*. Hence, in the Beauchemin Model, the persistence of migration carries on only for the predefined time *t*_pause _+ *t*_free_, after which it is immediately and completely lost. On the other hand, persistence in Fürth's equation gradually decays over time following an exponential term. This is why we previously used Fürth's equation to estimate the cell motility coefficient from short-term migration data [7]. However, whether this really leads to more accurate results remains to be determined.

A second notable consequence of the above result is that although the Beauchemin model has *three *microscopic parameters, its step-wise variance has only *two *degrees of freedom. Consequently, while the value of *t*_pause _would become apparent when analyzing the track of a single simulated cell at a very high resolution, it does no longer matter when a large population of cells that migrate asynchronously is analyzed, as shown by Proposition 12: Given a fixed *t*_free_, there are infinitely many combinations of *t*_pause _and *v*_free _that yield an identical motility coefficient and thereby an identical expected square displacement of the population. For example, the empirical mean square displacement of the best-fitting triplet (0.5, 2.0, 18.8) is identical (up to some rounding) to that of the triplet (9.32, 2.0, 40). Hence, we cannot expect to get reliable estimates for all three parameters *t*_free_*,t*_pause _and *v*_free _from fitting the model to mean square displacement data. This is consistent with the observation by Beauchemin et al. that the parameter *t*_pause _can always be set to 0 without much influence on the quality of the best fit.

**Figure 6:**
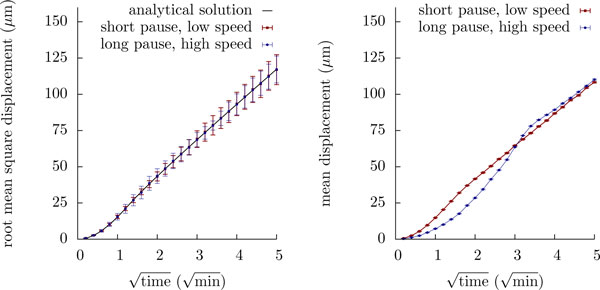
**Mean displacement can be more informative than mean square displacement**. The pause parameter of the Beauchemin model is not essential for the mean square displacement, but for the mean displacement. Left: Root mean square displacement curves generated for two populations with *t*_free _= 2 min and *t*_pause _= 0.5 min, *v*_free _= 18.8 *μ*m/min (red squares and error bars) or *t*_pause _= 9.32 min, *v*_free _= 40 *μ*m/min (blue circles and error bars). Simulations (N = 10000) with these two parameter yield the same results, as predicted by Proposition 12 (black line). Error bars show the standard error of the mean. Right: Although there is no difference with respect to the mean *square *displacement, the mean displacement curves of the two simulated populations differ.

However, Beauchemin et al. fitted their simulations to the *mean displacement *data, and the above result does not imply that the mean displacement too has just two degrees of freedom. Perhaps surprisingly, simulations show that this is in fact not the case (Figure 6): While mean square displacement plots generated with using the above parameter triplets and our analytical solutions are indeed consistent with each other, the mean displacement plots are significantly different. Hence, the mean displacement plot appears to preserve more information on the underlying migration process than does the mean square displacement plot, at least when applied to cell tracks simulated using the Beauchemin model.

### Confinement ratio

The confinement ratio is a measure of track straightness. In the context of lymphocyte migration analysis, it is also called "chemotactic index" or "meandering index" [2,3]. The confinement ratio is usually defined as the quotient of a path's displacement from the origin versus the complete length of the path. If the cell changes directions very often, the confinement ratio will tend to zero over time, while for directed migration without a random component, the confinement ratio is equal to 1.

Instead of the confinement ratio, we consider here the *squared *confinement ratio, which can be easily determined for the Beauchemin model from the previous result.

**Definition 13 **(Squared confinement ratio). *Consider a particle moving according to the Beauchemin model and let D*^2^(*t*) *denote the squared distance between the particle position at time t and its starting point. Then we define the squared confinement ratio C*^2^(*t*) *as follows:*

$$C^{2}\left(t\right)=\frac{D^{2}\left(t\right)}{t^{2}v_{free}^{2}}$$

By assuming *t*_pause _= 0 and applying the results from the previous section, we can determine the expected confinement ratio of the Beauchemin model as follows:

$$\begin{array}{llll} \text{E}\;\left[C^{2}\left(t\right)\right] & =\frac{1}{t^{2}v_{\text{free}}^{2}}\left[2Mt-2Mt_{\text{free}}\times \left\{\begin{array}{ccc} \frac{1}{3} & & t\geq t_{\text{free}}\\ \frac{1}{3} & {\left(\frac{t}{t_{\text{free}}}\right)}^{3}-{\left(\frac{t}{t_{\text{free}}}\right)}^{2}+\left(\frac{t}{t_{\text{free}}}\right) & t<t_{\text{free}} \end{array}\right)\right]\; & & \;\\ & =\frac{1}{t^{2}}\left[\frac{t_{\text{free}}}{3}t-\frac{t_{\text{free}}^{2}}{3}\times \left\{\begin{array}{ccc} \frac{1}{3} & & t\geq t_{\text{free}}\\ \frac{1}{3} & {\left(\frac{t}{t_{\text{free}}}\right)}^{3}-{\left(\frac{t}{t_{\text{free}}}\right)}^{2}+\left(\frac{t}{t_{\text{free}}}\right) & t<t_{\text{free}} \end{array}\right)\right]\; & & \;\\ & =\frac{t_{\text{free}}}{3t}-\frac{t_{\text{free}}^{2}}{3t^{2}}\times \left\{\begin{array}{ccc} \frac{1}{3} & & t\geq t_{\text{free}}\\ \frac{1}{3} & {\left(\frac{t}{t_{\text{free}}}\right)}^{3}-{\left(\frac{t}{t_{\text{free}}}\right)}^{2}+\left(\frac{t}{t_{\text{free}}}\right) & t<t_{\text{free}} \end{array}\right)\; & & \;\\ & =\frac{t_{\text{free}}}{3t}-\frac{1}{9}\times \left\{\begin{array}{cc} 1 & t\geq t_{\text{free}}\\ \frac{t}{t_{\text{free}}}-3+\frac{3t_{\text{free}}}{t} & t<t_{\text{free}} \end{array}\right)\; & & \;\\ & =\frac{t_{\text{free}}}{3t}-\frac{1}{9}\times \left\{\begin{array}{cc} 1 & t\geq t_{\text{free}}\\ \frac{3t_{\text{free}}^{2}+t^{2}}{t_{\text{free}}t}-3 & t<t_{\text{free}} \end{array}\right)\; & & \;\\ & =\ldots \; & & \;\\ & =\frac{1}{3\text{max}\left(t,t_{\text{free}}\right)}\left(t_{\text{free}}-\frac{t\;\times \;t_{\text{free}}}{3\text{max}{\left(t,t_{\text{free}}\right)}^{2}}\right)\; & & \;\\ & \; & \end{array}$$

Note that $\text{E}\;\left[C^{2}\left(0\right)\right]=\frac{1}{3}$, because the path length *tv*_free _is measured in three dimensions while the expected observed square distance is projected to one dimension.

Beltman et al. [3] have argued that the confinement ratio is not well suited for comparisons between different experiments because it tends to zero over time, and is therefore sensitive to the duration of the experiment from which it was estimated. They suggested to instead normalize the confinement ratio by multiplying it with the square root of time, such that it converges to a constant value. Applying this normalization to the squared confinement ratio of the Beauchemin model, we obtain

(11) $$t\;\times \;\text{E}\;\left[C^{2}\left(t\right)\right]\;=\;\frac{t}{3\text{max}\left(t,t_{\text{free}}\right)}\left(t_{\text{free}}-\frac{t\;\times \;t_{\text{free}}}{3\text{max}{\left(t,t_{\text{free}}\right)}^{2}}\right),$$

which for *t *≥ *t*_free _simplifies to

(12) $$t\;\times \;\text{E}\;\left[C^{2}\left(t\right)\right]\;=\;\frac{t_{\text{free}}\left(3t-1\right)}{9t}.$$

Thus, the normalized squared confinement ratio converges to *t*_free_/3. When there is evidence that the observed migration process really behaves roughly like the Beauchemin model, this could be a good rule of thumb for quick estimations of the persistence time from experimental data.

## Simulating biased migration

For our recent study of the detection limits of two-photon imaging [7], we extended the Beauchemin model for simulating three different types of biased migration, for which we used the term *taxis modes *[18] to indicate that such biased migration in cells usually occurs in response to an external stimulus. These three taxis modes were *orthotaxis*, where the movement speed is adjusted, *klinotaxis*, where the duration of the "free runs" is changed, and *topotaxis*, where the turning angle distribution of the simulated cell is changed. Thus, these modifications can be viewed as semi-mechanistic ones that attempt to describe ways in which a cell could re-adjust its migration behaviour from pure random migration to biased random migration in response to an external stimulus. Before we review these modifications, we start by presenting a fourth method to simulate biased migration which is not mechanistically motivated, but is much easier to analyze.

In all cases, taxis is spatially and temporarily uniform and controlled by two parameters: The vector $\vec{b}\in {\mathbb{R}}^{3}$, assumed to have unitary length, sets the direction of the taxis, and the parameter *p *∈ [0, 1] determines the magnitude of taxis, with *p *= 0 equivalent to an unbiased random walk and *p *= 1 to a maximally biased random walk; the precise meaning of the parameter *p *will be defined for each case below.

### Simple phenomenological model

A simple ad-hoc method to extend the Beauchemin model for simulating biased migration is the following: During the pause phase, the cell is no longer kept stationary, but moves into the bias direction $\vec{b}$ with speed *p *· *v*_free_. This model is very simple to analyze, because the modification is deterministic and so changes only the mean, but not the variance of each step. Hence, the motility coefficient remains the same.

**Proposition 14 **(Taxis speed for the simple model). *The length of the convection coefficient*, ||*C*||*, of the simple biased migration model is given by*

$$∥C∥=\frac{p\;\cdot \;v_{free}\;\cdot \;t_{pause}^{2}}{t_{free}+t_{pause}}.$$

*Proof*. During each pause phase, the cell moves a distance *p *· *v*_free _· *t*_pause _into the bias direction. ||*C*|| is then simply the product of this distance and the total fraction of time that the cell spends in the pause phase.    □

There might be some use for the simple biased migration model in situations where it is necessary to study the effect of bias independently of the motility coefficient. However, it is difficult to imagine how such a situation could arise biologically. Below we therefore discuss model modifications that have a more realistic motivation. For each of these modifications, it is possible to analytically derive the convection coefficient, i.e., the expected speed of the bias.

Throughout the rest of this section, we let the vector $\vec{d}$ denote the orientation vector of the Beauchemin model (i.e., a vector randomly sampled from the unit sphere), and $\vec{b}$ the bias direction. Moreover, we let $\left(\xi^{\prime },\;v^{\prime },\;\zeta^{\prime }\right)$ denote the vector of random variables representing a step of the modified model, while (*ξ*, *υ*, *ζ*) denotes a step in the original model.

### Orthotaxis model

In the orthotaxis model, the migration speed is no longer constant but depends on the chosen target direction. The more consistent this direction is with the target direction, the faster the simulated cell will move. Such bias could occur for instance in response to some external dragging force, and might be illustrated by imagining a random stroll through a city on a very windy day.

Formally, we define the adjusted speed as follows:

$$v_{\text{free}}^{\prime }\;=\;v_{\text{ free}}\left(1+p\;\langle \vec{b},\;\vec{d}\rangle \right)$$

Here 〈·,·〉 denotes the scalar product and *p *the bias strength parameter. Note that the movement perpendicular to $\vec{b}$is not affected at all, while movement against the direction $\vec{b}$ is slower and in direction $\vec{b}$ it is faster. More precisely, the relation between *p *and the bias speed *v*_taxis _is given by the following proposition.

**Proposition 15 **(Speed of orthotaxis [7]). *The convection coefficient *||*C*|| *of the orthotaxis model is given by*

$$∥C∥\;=\;v_{free}\cdot \;\frac{p\;t_{free}}{3\left(t_{free}+\;t_{pause}\right)}.$$

*Proof*. We can assume without loss of generality that the bias is along the first dimension, i.e. $\vec{d}\;=\;\left(1,\;0,\;0\right)$, and thus $\langle \vec{b},\vec{d}\rangle \;=\;\xi$. We are interested in the expectation $\text{E}\;\left[\xi^{\prime }\right]$; it is easy to see that the expectations $\text{E}\;\left[v^{\prime }\right]$ and $\text{E}\;\left[\zeta^{\prime }\right]$ are still 0. Then we get

$$\begin{array}{llll} \xi^{\prime } & =\;{v^{\prime }}_{\text{free}}\xi =v_{\text{free}}\left(1+p\xi \right)\xi \; & & \;\\ & =v_{\text{free}}\left(\xi +p\xi^{2}\right).\; & & \;\\ & \; & \end{array}$$

Because *ξ *is uniformly distributed on [−1, 1] (see proof of Proposition 10), the expected location $\text{E}\;\left[\xi^{\prime }\right]$ at the end of the step is

$$\begin{array}{llll} \text{E}\;\left[\xi^{\prime }\right]\; & =\;\text{E}\;\left[v_{\text{ free}}\cdot \;t_{\text{free}}\left(\xi +p\xi^{2}\right)\right]\; & & \;\\ & =v_{\text{free}}\cdot \;t_{\text{free}}\left(\text{E}\;\left[\xi \right]\;+\;p\text{E}\;\left[\xi^{2}\right]\right)\;\; & & \;\\ & =\;v_{\text{free}}\cdot \;t_{\text{free}}\cdot \;p/3.\; & & \;\\ & \; & \end{array}$$

Dividing this expected location $\text{E}\;\left[\xi^{\prime }\right]$ by the step duration *t*_free _+ *t*_pause _yields the claimed expression for ||*C*||.    □

### Topotaxis model

In the case of topotaxis, the cell adjusts its turning behaviour but keeps the speed constant. This also affects both the variance and the mean of the cell step distribution. Topotaxis could be a likely bias mechanism for cells migrating on some sort of structural cell network in the tissue, like it was suggested to be the case for T cells in secondary lymphoid organs [19].

We implement topotaxis by skewing the distribution from which we pick the cell's orientation $\vec{d}\;=\;\left(\xi ,\;v,\;\zeta \right)$. Recall that this is a uniform distribution on a sphere in the unbiased case. Such a distribution can also be generated by sampling *ξ *uniformly from [−1, 1] and then picking *υ*, *ζ *uniformly from a circle with radius $\sqrt{1-\xi^{2}}$. Thus, the probability density function *f*_ξ_(*x*) is defined as follows:

$$f_{\xi }\left(x\right)\;=\;\frac{1}{2}\text{ , }\;\;x\in \left[-1,1\right].$$

For the modified model version, we define a parameterized version of *f_ξ _*(*x*) as follows:

$$f_{\xi^{\prime }}\left(x\right)\;=\;\frac{1+px}{2}\text{ , }\;x\in \left[-1,1\right].$$

Hence, for *p *= 0 we obtain the distribution  of the unbiased case, while for *p *= 0.5 we get the slightly skewed distribution  and for *p *= 1 we obtain the maximally skewed distribution .

This modification of the Beauchemin model yields the same convection coefficient as in the previous case.

**Proposition 16 **(Speed of topotaxis [7]). *The speed *||*C*|| *of the directional motion induced by the topotaxis model is given by the equation*

$$∥C∥\;=\;v_{free}\cdot \;\frac{p\;t_{free}}{3\left(t_{free}+\;t_{pause}\right)}.$$

*Proof*. This is simply shown by calculating $\text{E}\;\left[\xi^{\prime }\right]$ for the probability density function defined above.    □

### Klinotaxis model

Lastly, we can also induce bias by modifying the duration *t*_free _of the free run depending on the picked orientation $\vec{d}$. A well-known biological example of such bias is the "run-and-tumble" motion by which *E. Coli *searches for food: Because the bacterium is too small to sense concentration gradients *in situ*, it is capable of comparing concentration measurements along a persistent path until it figures out whether it is going in the right direction. When it senses that it is moving in the right direction (run), it will try to keep going, while otherwise it will stop its motion soon (tumble) and orient itself in a new random direction.

We implement klinotaxis by modifying the free run duration *t*_free _depending on the angle between $\vec{d}$ and $\vec{b}$ as follows:

$$t_{\text{free}}^{\prime }=\;t_{\text{ free}}\left(1+p\langle \vec{b},\;\vec{d}\rangle \right)$$

Again, this modification leads to exactly the same bias speed as found for the previous two models.

**Proposition 17 **(Speed of klinotaxis [7]). *The speed *||*C*|| *of the directional motion induced by the klinotactic modification of the Beauchemin model is given by the equation*

$$∥C∥\;=\;v_{free}\cdot \;\frac{p\;t_{free}}{3\left(t_{free}+\;t_{pause}\right)}.$$

*Proof*. The proof follows along the lines of a similar model of *E. Coli *run-and-tumble motion discussed by de Gennes [20]. We start again by analyzing a single free run of a cell projected onto the direction of bias, which is assumed without loss of generality to be along the first dimension *ξ*. Again we need to determine $\text{E}\;\left[\xi^{\prime }\right]$. We start by writing $\xi^{\prime }$ as

$$\xi^{\prime }=v_{\text{ free}}\cdot \;\xi \;\cdot \;t_{\text{free}}\cdot \;\left(1+p\xi \right)\;.$$

Now let us assume for a moment that *t*_pause _= 0. It is easy to verify that $\text{E}\;\left[t_{\text{free}}^{\prime }\right]=t_{\text{free}}$. Thus, the average speed towards the bias direction over many successive steps is $\text{E}\;\left[\xi^{\prime }/t_{\text{free}}\right]$. A simular calculation as in the proof of Proposition 15 yields

$$\text{E}\left[\xi^{\prime }/t_{\text{ free}}\right]\;=\;v_{\text{free}}\;\cdot \;p/3.$$

Now we arrive at the claimed expression by multiplying the above with the overall fraction of time that the particle spends in the free run phase, which is

$$t_{\text{free}}/\left(t_{\text{free}}+\;t_{\text{pause}}\right).$$

   □

Note that the klinotaxis model no longer fits within our mathematical framework because the duration of a step in the model is no longer constant. Thus, the central limit theorem we applied in that section no longer applies, and a different central theorem would be needed to show formally that the model converges to a Brownian motion.

**Figure 7:**
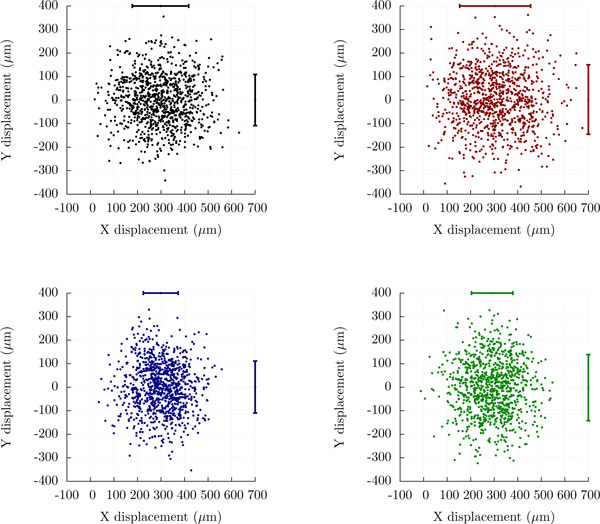
**Taxis skews the random motility component**. Displacement of *in silico *cells when applying the four different methods of simulating biased migration along the X axis using the methods discussed in this paper, i.e., the simple phenomenological model (black), orthotaxis (red), topotaxis (blue), and klinotaxis (green). For each panel, *N *= 1000 cells were simulated using the parameters *t*_pause _= 0.5 min, *t*_free _= 2.0 min, and *v*_free _= 18.8 *μ*m/min. Cell displacements are shown after one hour of simulation time, and the taxis parameters *p *are in each case set such that the expected displacement is 300 *μ*m per hour. The length of the scale bars is proportional to the dimension-wise variances of the cell displacements, demonstrating that these variances change when biased migration is introduced.

Moreover, we point out that for all modifications discussed in this section except for the simple phenomenological one, the random motility component changes. Therefore, these modifications cause as a side-effect a modified motility coefficient. In general, the random motility component are even affected in a non-isotropic fashion, such that the motility coefficient would need to be described using a 3 × 3 matrix instead of a single scalar value. In Figure 7, we show some simulation results that illustrate this point.

## Conclusions

In this article, we have compiled both old and new analytical results on the Beauchemin model of lymphocyte migration, which was originally a model of purely random motility, but is now able to accommodate partially biased migration as well. An important practical consequence of these results is that validation of the model against experimentally determined motility coefficient, mean square displacement data, and confinement ratio curves does no longer need to be performed by simulation, because these quantities can be calculated directly from the model parameters. This also allows for the use of standard numerical fitting procedures, such as implemented in popular computer algebra packages, to estimate the model parameters from such data by least-squares-fitting. An important observation was that when simulating a large population of unsynchronized cells, the model is equivalent to the ideal chain model of polymer physics, which is however only true with respect to the quantities named above and not for others like the mean displacement. Interestingly, this shows that we can sometimes infer information from mean displacement data that we cannot infer from mean *square *displacement data. However, an analytical derivation of the mean displacement of the model has not yet been achieved, and can be expected to be complicated judging from the experiences with similar models [21]. For now, we leave this as an important open problem.

Subtle biased migration of lymphocytes can point to important biological functions or phenomena [22,23], and the use of mathematical models has helped to understand the quantitative impact of such effects [23,7]. The modifications discussed above facilitate simulations of such subtle biases with pre-defined speed, again removing the need for fitting the bias strength parameter to data by using simulations. However, all but one of the discussed modifications also affect the random migration component, which is likely also true in biological scenarios where randomly migrating cells respond to an external stimulus. Therefore, another important task that remains to be solved is to analytically derive the motility coefficient matrices for the topotaxis, klinotaxis, and orthotaxis models.

## Competing interests

The authors declare that they have no competing interests.

## Authors' contributions

JT conceived the study, participated in the mathematical analysis, and wrote the paper; MS participated in the mathematical analysis and helped write the paper; RJdB participated in the study conception and helped write the paper.

## Declarations

This article has been published as part of *BMC Bioinformatics *Volume 14 Supplement 6, 2013: Selected articles from the 10th International Conference on Artificial Immune Systems (ICARIS). The full contents of the supplement are available online at http://www.biomedcentral.com/bmcbioinformatics/supplements/14/S6.
